# Global research landscape and emerging trends of tertiary lymphoid structures in autoimmune diseases: a bibliometric analysis

**DOI:** 10.3389/fimmu.2026.1864891

**Published:** 2026-07-14

**Authors:** Jiaqi Wang, Yiyuanzi Zhao, Xixuan Chen, Tianshu Dong, Zhiyi Feng, Zhongmian Zhang, Zhihong Li

**Affiliations:** 1 Dongzhimen Hospital, Beijing University of Chinese Medicine, Beijing, China; 2 Beijing Hospital of Traditional Chinese Medicine Affiliated to Capital Medical University, Beijing, China

**Keywords:** autoimmune diseases, B cells, bibliometric analysis, chemokine networks, immune microenvironment, tertiary lymphoid structures

## Abstract

**Background:**

Tertiary lymphoid structures (TLSs) are increasingly regarded as important local immune niches in chronic inflammation and autoimmunity. However, a focused bibliometric overview of TLS research in autoimmune diseases is still lacking.

**Methods:**

Publications on TLSs in autoimmune diseases published between 2006 and 2025 were retrieved from the Web of Science Core Collection and Scopus databases. After screening and deduplication, the eligible records were analyzed using Bibliometrix/Biblioshiny, VOSviewer, and CiteSpace to assess publication trends, collaboration patterns, co-citation networks, keyword clustering, and thematic evolution.

**Results:**

A total of 479 publications were included. Annual output showed an overall upward trend, indicating sustained interest in this field. The United States occupied a central position in the international collaboration network, while several European countries also made important contributions. Rheumatoid arthritis, Sjögren’s syndrome, systemic lupus erythematosus/lupus nephritis, and myasthenia gravis emerged as the major disease contexts of TLS research. The field evolved from early attention to lymphoid neogenesis, chemokine-mediated tissue organization, and ectopic germinal center-like responses toward local B-cell hyperactivity, disease-specific immune microenvironments, and clinical heterogeneity. Recent frontiers include T follicular helper/peripheral helper T-cell axes, stromal remodeling, fibroblast-associated immune organization, disease subsets, and single-cell/spatial omics approaches.

**Conclusion:**

TLS research in autoimmune diseases has evolved from structural recognition toward functional interpretation, disease stratification, and translational exploration. Further studies integrating mechanistic investigation with clinical stratification are needed to clarify the translational relevance of TLSs in precision medicine.

## 1 Introduction

Autoimmune diseases are a group of chronic inflammatory disorders driven by the breakdown of immune tolerance and persistent immune activation, which can involve multiple organs, including the joints, exocrine glands, kidneys, central nervous system, and thymus, and are characterized by marked heterogeneity and tissue-specific injury (1–4). In addition to systemic immune dysregulation, the persistent local immune cell infiltration and chronic inflammatory microenvironment within target organs suggest that the organization and maintenance of local immune responses also play important roles in disease persistence and progression (5–7).

Tertiary lymphoid structures (TLSs), also referred to in some studies as tertiary lymphoid organs (TLOs) or ectopic lymphoid structures (ELSs), are locally organized lymphoid aggregates that develop in non-lymphoid tissues under conditions of persistent antigenic stimulation, chronic inflammation, or tissue injury and exhibit features resembling those of secondary lymphoid organs (8). TLSs are typically characterized by the segregation of T-cell zones and B-cell follicles and may be accompanied by high endothelial venules, follicular dendritic cell networks, and germinal center-like reactions, thereby supporting sustained local lymphocyte recruitment, antigen presentation, and humoral immune responses (9–11). In tumors, TLSs are generally associated with more favorable antitumor immunity and better prognosis (12–14), whereas in autoimmune diseases, they are more commonly regarded as important microenvironments that promote persistent local immune activation, autoantibody production, and amplification of tissue injury (3).

In recent years, research on TLSs in a variety of autoimmune diseases has accumulated steadily, with particularly important advances in rheumatoid arthritis, Sjögren’s syndrome, systemic lupus erythematosus/lupus nephritis, and myasthenia gravis. Previous studies have shown that TLSs not only exhibit well-defined organizational features, but also support local B-cell maturation, autoantibody production, and the persistence of disease-related immune responses. Meanwhile, the development of chemokine network studies, helper T-cell subset profiling, stromal cell remodeling research, and single-cell and spatial omics technologies is driving TLS research from structural observation toward mechanistic investigation and disease stratification.

It should be noted that a bibliometric study published in 2024 has already examined global TLS research trends (15). However, it was based on the WoSCC as a single database, covered the period from 2014 to 2023, and its research hotspots were predominantly driven by cancer- and immunotherapy-related topics. Therefore, that study provided an overall overview of the TLS field rather than a focused analysis of the autoimmune disease setting.

Based on this, the present study performed a bibliometric analysis of TLS-related research in autoimmune diseases using the Web of Science Core Collection and Scopus databases, covering the period from 2006 to 2025. By integrating Bibliometrix/Biblioshiny, VOSviewer, and CiteSpace, we systematically evaluated annual publication trends, international collaboration patterns, core authors and institutions, knowledge bases, research hotspots, and thematic evolution in this field, with the aim of elucidating the developmental trajectory, key issues, and emerging frontiers of TLS research from the perspective of autoimmune diseases, and of providing a reference for subsequent mechanistic studies and clinical translation.

## 2 Methods

### 2.1 Data sources and search strategy

This study was designed as a bibliometric analysis. Data were retrieved from the Web of Science Core Collection (WoSCC) and Scopus databases, and the detailed search strategies are presented in **Table 1**. To minimize potential bias caused by dynamic database updates, both databases were searched on the same day, namely March 3, 2026. The retrieval period was limited from January 2006 to December 2025. Only English-language publications were included, and the document types were restricted to articles and reviews.

**Table 1:** Search strategies used in the Web of Science Core Collection and Scopus databases.

Database	Search query
WoSCC	TS=(“tertiary lymphoid structure*” OR “tertiary lymphoid organ*” OR “ectopic lymphoid structure*” OR “ectopic lymphoid organ*” OR “ectopic lymphoid tissue” OR “ectopic lymphoid neogenesis” OR “tertiary lymphoid tissue” OR “lymphoid neogenesis” OR (“lymphoid aggregate*” NEAR/3 (ectopic OR tertiary)) OR (“germinal center*” NEAR/3 ectopic) OR “ectopic germinal center*”) AND TS=(autoimmun* OR “auto-immune” OR (“autoimmune” NEAR/2 (disease* OR disorder* OR condition*))) NOT TS=(cancer OR tumor OR tumor OR neoplasm* OR carcinoma* OR oncology)
Scopus	TITLE-ABS-KEY (“tertiary lymphoid structure*” OR “tertiary lymphoid organ*” OR “ectopic lymphoid structure*” OR “ectopic lymphoid organ*” OR “ectopic lymphoid tissue” OR “ectopic lymphoid neogenesis” OR “tertiary lymphoid tissue” OR “lymphoid neogenesis” OR ((“lymphoid aggregate*” W/3 (ectopic OR tertiary))) OR ((“germinal center*” W/3 ectopic)) OR “ectopic germinal center*”) AND TITLE-ABS-KEY (autoimmun* OR “auto-immune” OR (autoimmune W/2 (disease* OR disorder* OR condition*))) AND NOT TITLE-ABS-KEY (cancer* OR tumor* OR tumor* OR neoplasm* OR malignant* OR carcinoma* OR adenocarcinoma* OR sarcoma* OR lymphoma* OR leukemia* OR leukemia* OR “multiple myeloma” OR myeloma* OR “oncology*” OR “metast*” OR “chemotherapy*” OR “radiotherapy*” OR immunotherapy*)

### 2.2 Literature screening and data extraction

The inclusion criteria were English-language original articles and reviews primarily focusing on TLS-related research in autoimmune diseases. The exclusion criteria included conference abstracts, editorials, letters, book chapters, news reports, and publications unrelated to the research topic. Records related to non-autoimmune chronic inflammatory conditions or other irrelevant TLS contexts were excluded after title and abstract screening.

Because some autoimmune diseases, particularly Sjögren’s syndrome, may involve lymphoproliferative tendency or lymphoma risk, studies conducted in autoimmune disease settings were retained when their primary focus remained autoimmune pathology and TLS-related immune microenvironments. In contrast, studies primarily centered on overt lymphoma, hematologic malignancy, or oncology-oriented mechanisms and treatments were excluded.

**Figure 1:**
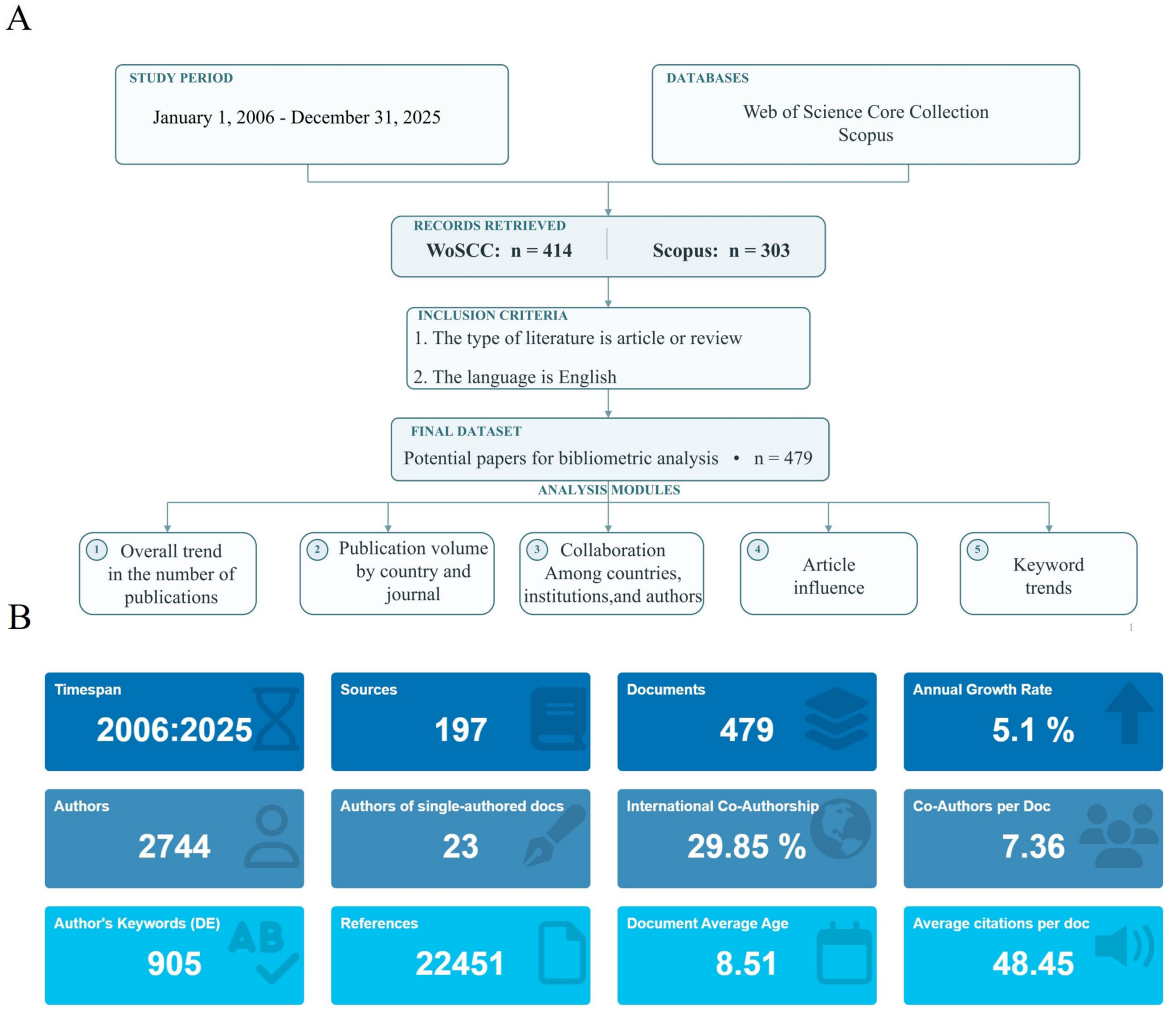
Literature retrieval, screening workflow, and basic bibliometric characteristics of the included publications. **(A)** Flowchart of literature retrieval and screening, including study period, databases searched, number of records retrieved, inclusion criteria, final dataset, and analytical modules. **(B)** Summary of the basic bibliometric indicators of the included publications, including timespan, number of sources, documents, annual growth rate, authors, single-authored documents, international co-authorship, co-authors per document, authors’ keywords, references, document average age, and average citations per document.

After retrieval, records from WoSCC and Scopus were merged and deduplicated using a three-step procedure based on DOI matching, title matching, and manual verification of ambiguous records. After deduplication, title and abstract screening was independently conducted by two reviewers. Any disagreements were resolved through discussion and consensus. The final dataset used for bibliometric analysis was established after this screening procedure. The literature screening process is shown in **Figure 1**.

The extracted information included complete bibliographic data, such as title, authors, institutions, countries, abstracts, keywords, journals, publication year, and cited references, for subsequent analyses.

### 2.3 Bibliometric analysis

Bibliometric analysis and visualization were performed using Bibliometrix/Biblioshiny in the R environment (version 4.3.3), VOSviewer (version 1.6.20), and CiteSpace (version 6.3.R1). Bibliometrix/Biblioshiny was used to analyze annual publication trends, source journals, author productivity, corresponding author countries, and three-field plots. VOSviewer was used to construct collaboration networks among countries, institutions, and authors, as well as keyword co-occurrence and density maps. CiteSpace was used for co-citation analysis, clustering, burst detection, and timeline visualization to identify research hotspots, knowledge bases, and thematic evolution.

In CiteSpace, the time slicing was set from 2006 to 2025, with 1 year per slice. The selection criteria were set to g-index (k = 15), and the pruning method was set to pruning sliced networks. Other parameters were adjusted according to the analytical requirements of specific visualizations.

## 3 Results

### 3.1 Literature retrieval and basic characteristics

According to the predefined search strategy, a total of 717 relevant publications were initially retrieved from the Web of Science Core Collection and Scopus databases, including 414 records from WoSCC and 303 records from Scopus. After deduplication and screening, 479 publications were included in the final bibliometric dataset (**Figure 1A**). Further bibliometric profiling showed that these publications were distributed across 197 journals and involved 2,744 authors, including 23 authors of single-authored papers (**Figure 1B**). The annual growth rate of the field was 5.1%, the proportion of internationally co-authored publications was 29.85%, and the average number of authors per document was 7.36. In addition, the included publications contained 905 author keywords and 22,451 cited references, with a mean document age of 8.51 years and an average of 48.45 citations per document. Overall, this field has developed a relatively stable research foundation and exhibits sustained growth and a high degree of collaboration.

### 3.2 Annual publication trends and characteristics of country/region distribution

**Figure 2:**
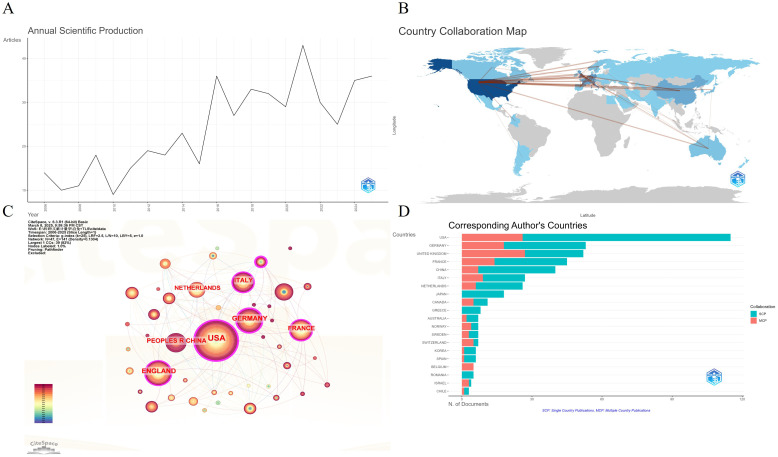
Annual publication trend and country/region distribution in research on tertiary lymphoid structures in autoimmune diseases. **(A)** Annual scientific production from 2006 to 2025. **(B)** Global map of international collaboration among countries/regions. **(C)** Country/region collaboration network generated by CiteSpace. Node size represents publication output, and links indicate collaborative relationships. **(D)** Distribution of corresponding author’s countries, showing single-country publications (SCP) and multiple-country publications (MCP).

As shown in **Figure 2A**, the annual number of publications in this field generally exhibited a fluctuating upward trend over the study period. From 2006 to 2014, annual publication output remained at a relatively low level and increased slowly. After 2015, publication volume increased markedly, indicating that the field had entered a phase of more rapid development. Although some year-to-year fluctuations were observed thereafter, annual output remained at a relatively high level overall and reached a stage-specific peak around 2021, suggesting sustained academic interest in this research area.

The international collaboration pattern is shown in **Figure 2B**. A relatively well-defined global country collaboration network has formed, with the United States occupying the central position and maintaining extensive links with multiple countries in Europe and the Asia-Pacific region, reflecting strong cross-regional collaborative capacity. **Figure 2C** further shows that the United States, China, Germany, Italy, France, the United Kingdom, and the Netherlands had relatively large nodes and dense connections, indicating that these countries were major participants and important hubs in the international collaboration network. The overlay visualization also suggests that earlier active countries were mainly concentrated in the core research groups of Europe and North America, whereas some Asian and other countries have gradually entered the network in recent years, indicating that international participation in this field is expanding, although knowledge production remains concentrated in a limited number of high-impact countries.

**Table 2** and **Figure 2D** jointly present the distribution and collaboration patterns of corresponding authors’ countries. The United States ranked first with 115 articles, accounting for 24.0% of the total dataset, including 89 single-country publications (SCP) and 26 multiple-country publications (MCP), and remained the leading contributor in this field. Germany (53 articles) and the United Kingdom (52 articles) followed closely, while France (45 articles) and China (40 articles) also ranked among the major contributors. Most countries were dominated by SCP, although the degree of international collaboration varied substantially across countries. The United Kingdom showed the highest MCP proportion (51.9%), indicating the strongest international collaborative profile. Canada (45.5%), Germany (34.0%), Italy (33.3%), and France (31.1%) also showed relatively high levels of cross-national collaboration, whereas China (17.5%) and Japan (0%) relied more heavily on domestic research capacity.

**Table 2:** Corresponding author countries and collaboration patterns in TLS research on autoimmune diseases.

Country	Articles	Articles %	SCP	MCP	MCP %	TC	Average article citations
USA	115	24	89	26	22.6	6317	49.7
GERMANY	53	11.1	35	18	34	3278	60.7
UNITED KINGDOM	52	10.9	25	27	51.9	3177	61.1
FRANCE	45	9.4	31	14	31.1	2687	59.7
CHINA	40	8.4	33	7	17.5	938	22.9
ITALY	27	5.6	18	9	33.3	2959	105.7
NETHERLANDS	26	5.4	20	6	23.1	1431	55
JAPAN	18	3.8	18	0	0	592	26.9
CANADA	11	2.3	6	5	45.5	726	55.8
GREECE	8	1.7	8	0	0	214	26.8

SCP, single-country publications; MCP, multiple-country publications; TC, total citations.

In terms of citation impact, the United States had the highest total citation count (6,317), indicating the strongest overall academic influence. Germany, the United Kingdom, and France also showed high total citation counts, with 3,278, 3,177, and 2,687 citations, respectively. Notably, although Italy contributed a relatively smaller number of publications (27 articles), it had the highest average citations per article (105.7), indicating strong research quality and academic impact. The United Kingdom (61.1 citations/article), Germany (60.7 citations/article), France (59.7 citations/article), and Canada (55.8 citations/article) also demonstrated relatively high average citation impact, whereas China showed a comparatively lower average citation rate (22.9 citations/article).

Overall, this field has developed an international research landscape centered on the United States, with strong participation from several European countries. The United States maintained a dominant position in research output, collaboration networks, and academic influence, whereas European countries such as the United Kingdom, Germany, France, and Italy played important roles in international collaboration and high-quality research output. Meanwhile, Asian countries, particularly China, have become important contributors to this field, although there is still room for improvement in terms of international collaboration depth and citation impact.

### 3.3 Institutional thematic clustering, source journal dynamics, and the knowledge structure of core authors

**Figure 3:**
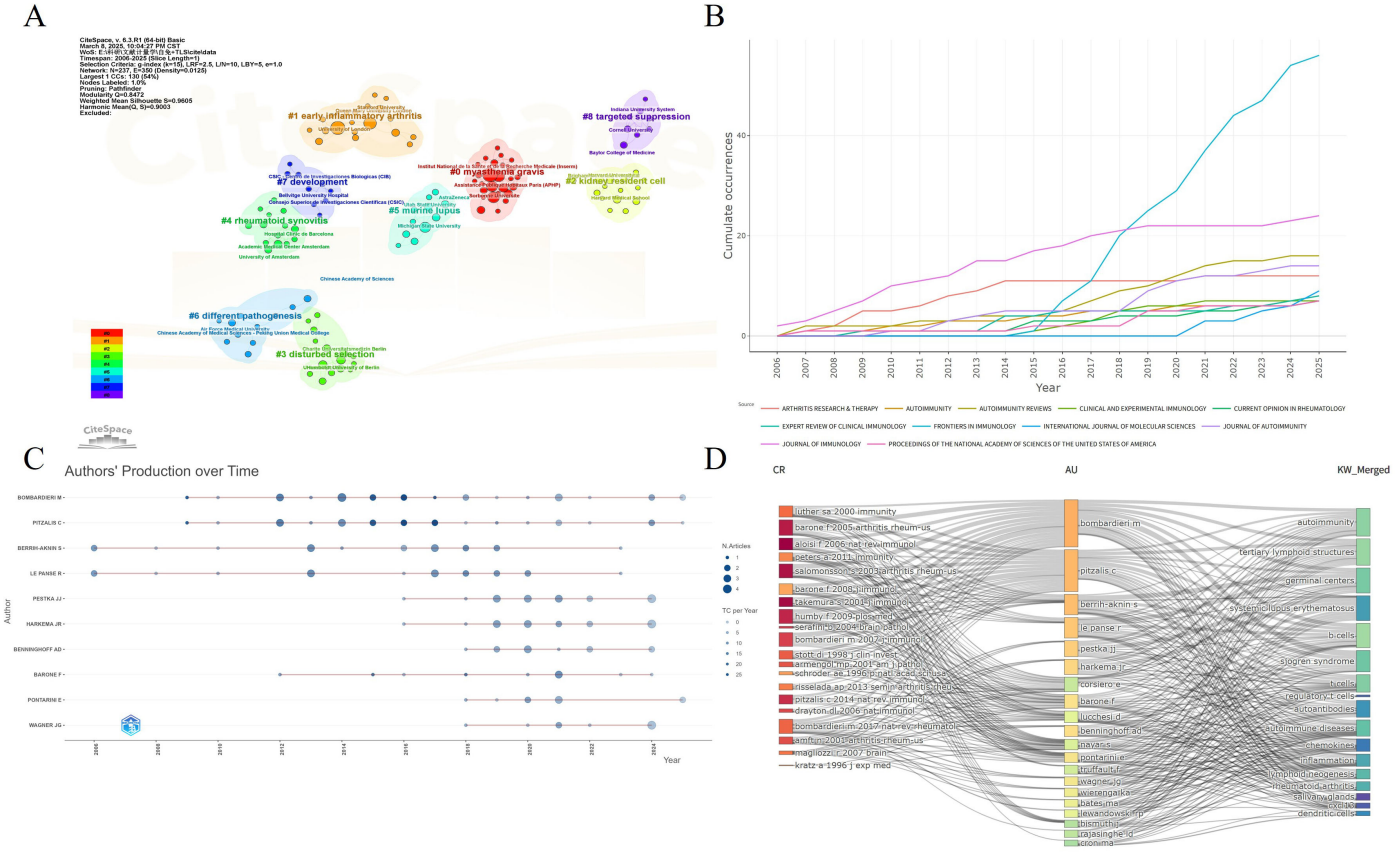
Institutional thematic clustering, source journal dynamics, and core author knowledge structure. **(A)** Institutional clustering map showing major thematic groups in the field. **(B)** Cumulative publication occurrences of major source journals over time. **(C)** Production of core authors over time. Bubble size represents the number of publications, and color intensity reflects citation impact per year. **(D)** Three-field plot showing the relationships among cited references (CR), authors (AU), and merged keywords (KW_Merged).

As shown in **Figure 3A**, the institutional clustering analysis generated a thematic structure with relatively clear boundaries, and the clustering results demonstrated good stability and consistency (Q = 0.8047, S = 0.9605). According to the cluster labels, the major research themes were primarily centered on myasthenia gravis, early inflammatory arthritis, rheumatoid synovitis, lupus, and targeted suppression. Overall, earlier studies focused more on the formation of ectopic lymphoid structures and the pathological basis of autoimmunity, whereas more recent studies have gradually shifted toward organ-specific immune injury and targeted regulation, suggesting that this field is evolving from mechanistic description toward more refined disease stratification and intervention-oriented exploration.

As shown in **Figure 3B**, the cumulative publication trajectories differed among journals. Among them, Frontiers in Immunology showed a more pronounced increase after 2015 than the other journals, highlighting its importance in this field. At the same time, the publishing platforms for this topic have expanded from traditional immunology journals toward journals related to clinical immunology and rheumatology, reflecting a continuous shift of this field toward clinical relevance and translational application.

The temporal distribution of core author productivity is shown in **Figure 3C**. Authors such as Bombardieri M, Pitzalis C, Berrih-Aknin S, and Le Panse R remained active over a relatively long period and constituted a stable core research group in this field. Meanwhile, new authors have continued to enter the field in recent years, indicating that this research direction has maintained strong academic vitality while preserving continuity.

**Figure 3D** shows a relatively concentrated knowledge linkage among highly cited references, core authors, and keywords. The main research trajectory was centered on autoimmunity, tertiary lymphoid structures, germinal centers, and topics such as systemic lupus erythematosus and Sjögren’s syndrome, indicating that the current research focus has moved beyond the structural phenomenon itself toward the organizational patterns and immunological significance of TLSs in autoimmune diseases.

### 3.4 Multilevel collaboration networks and the co-citation knowledge base

**Figure 4:**
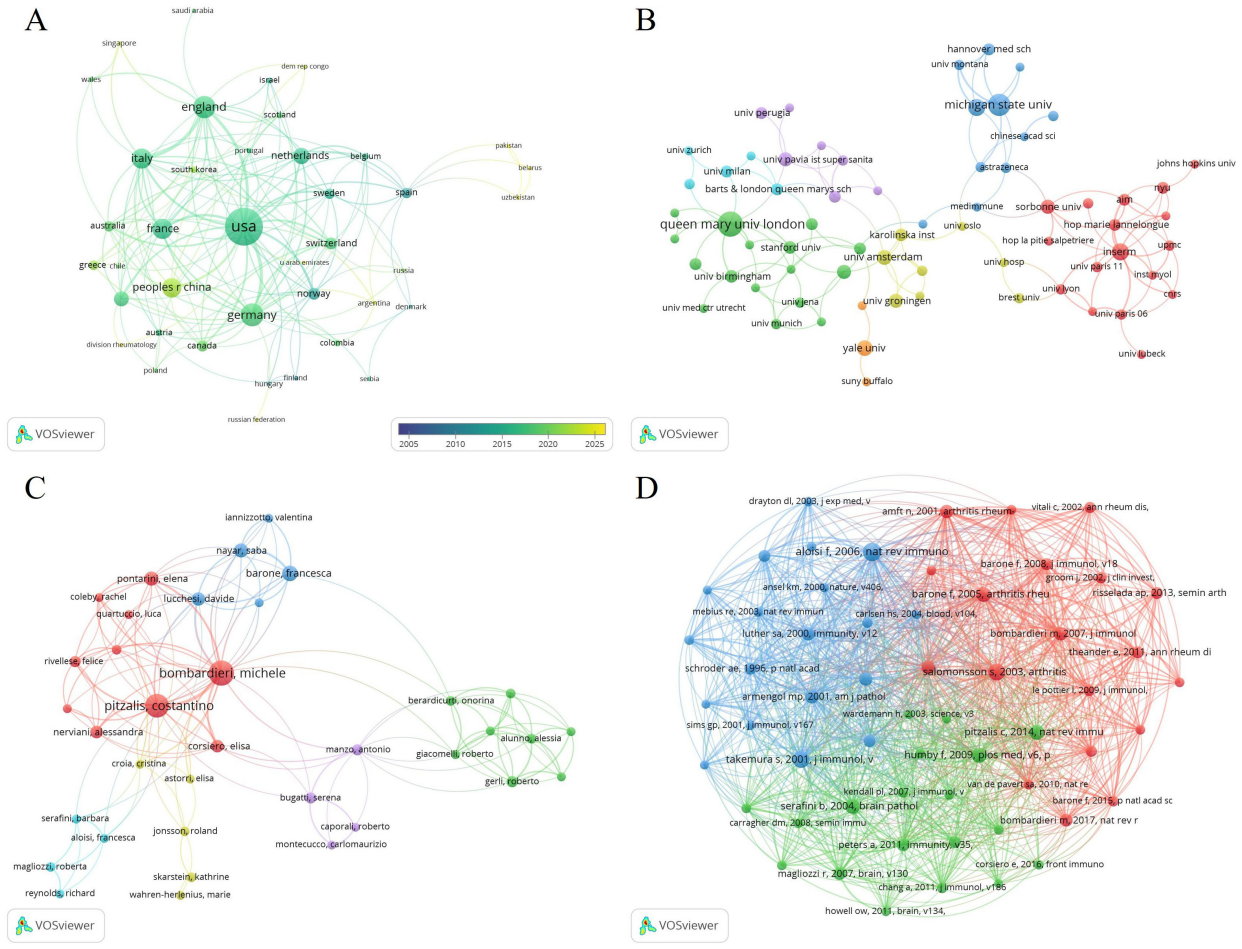
Collaboration networks at the country, institution, and author levels, and the co-cited reference network. **(A)** Overlay visualization of country collaboration network. Node color reflects the average publication year. **(B)** Overlay visualization of institutional collaboration network. **(C)** Author collaboration network showing major research teams and collaborative structures. **(D)** Co-cited reference network showing the intellectual base of the field.

As shown in **Figure 4A**, the country collaboration network exhibited a structure centered on the United States, with broad participation from multiple European countries. The United States, Germany, France, Italy, the United Kingdom, and the Netherlands had relatively large nodes and dense connections, identifying them as the major hubs of international collaboration in this field. The overlay visualization further showed that the earlier active countries were mainly concentrated in the core research groups of Europe and North America, whereas some Asian and other countries have gradually entered the network in recent years. This finding suggests that the scope of international participation in this field is expanding, although knowledge production remains concentrated in a limited number of high-impact countries.

The institutional collaboration network is presented in **Figure 4B**. Overall, inter-institutional collaboration formed several relatively stable subnetworks, among which Queen Mary University of London, INSERM, the University of Amsterdam, and Michigan State University occupied relatively important positions and connected different collaborative groups. These results indicate that current research is not distributed homogeneously; rather, it is organized as a multicenter collaborative pattern supported by a limited number of core institutions. Different institutional groups maintain their own research continuity while also showing a certain degree of cross-network interaction.

The author collaboration network is shown in **Figure 4C**. Bombardieri Michele and Pitzalis Costantino were located at the core of the network and were surrounded by closely connected collaborators, forming one of the most active collaborative units in this field. At the same time, authors such as Gerli Roberto formed relatively independent but internally cohesive subgroups. Overall, author collaboration displayed a clear team-based pattern, and core authors not only contributed sustained research output but also, to some extent, shaped the research directions and collaborative structure of the field.

**Figure 4D** shows that the co-cited reference network had a high degree of aggregation and formed several closely connected knowledge clusters. Frequently co-cited references were mainly concentrated in the representative studies of authors such as Barone, Bombardieri, Aloisi, Humby, and Peters, suggesting that these publications constitute an important knowledge base of the field. In combination with the network structure, the co-citation relationships were mainly centered on ectopic lymphoid structures, local germinal center responses, and autoimmune inflammatory microenvironments, indicating that the core knowledge of this field is not simply fragmented, but is instead built upon a group of interrelated landmark studies and continuously extended by subsequent research.

### 3.5 Highly cited references, citation bursts, and the evolution of co-citation clusters

As shown in **Table 3**, the highly cited publications in this field were mainly concentrated on TLSs, local B-cell responses, and mechanisms related to autoimmune diseases. Among them, the review by Aloisi F published in Nature Reviews Immunology in 2006 had the highest global citation count (774) and also ranked among the top in local citations, indicating that it not only exerted broad influence but was also deeply embedded in the knowledge system of this field. The study by Humby F published in PLOS Medicine in 2009 and the study by Chang A published in the Journal of Immunology in 2011 also showed high local citation frequencies, suggesting that research focused on local germinal center-like responses and renal or target-organ immune structures has become an important intellectual foundation of this field. By contrast, some publications with high global citations but relatively low local citations more likely represent peripheral influences related to this topic rather than core components of the main knowledge chain.

**Table 3:** Most influential references in research on tertiary lymphoid structures in autoimmune diseases according to citation indicators.

DOI	Year	Global citations	Local citations	LC/GC ratio (%)	Normalized local citations	Normalized global citations
10.1038/nri1786	2006	774	84	10.85	7.69	6.82
10.1523/JNEUROSCI.1114-16.2016	2016	649	0	0.00	0.00	7.97
10.1016/j.rmed.2018.12.015	2019	416	0	0.00	0.00	8.06
10.1371/journal.pmed.0060001	2009	400	50	12.50	7.50	4.03
10.1681/ASN.2013010026	2013	400	1	0.25	0.39	4.91
10.1016/j.it.2012.04.006	2012	344	0	0.00	0.00	5.11
10.1016/j.immuni.2008.05.010	2008	338	1	0.30	0.26	3.99
10.1016/j.it.2009.06.003	2009	332	4	1.20	0.60	3.34
10.1084/jem.20080752	2009	327	13	3.98	1.95	3.29
10.1186/ar4555	2014	295	5	1.69	1.85	4.37
10.4049/jimmunol.1001983	2011	287	27	9.41	7.79	4.22
10.1038/nrrheum.2018.1	2018	279	12	4.30	4.95	5.59
10.1016/j.cytogfr.2013.03.001	2013	238	0	0.00	0.00	2.92
10.1186/ar3433	2011	211	0	0.00	0.00	3.10
10.1038/nrrheum.2016.217	2017	209	38	18.18	13.50	3.91

**Figure 5:**
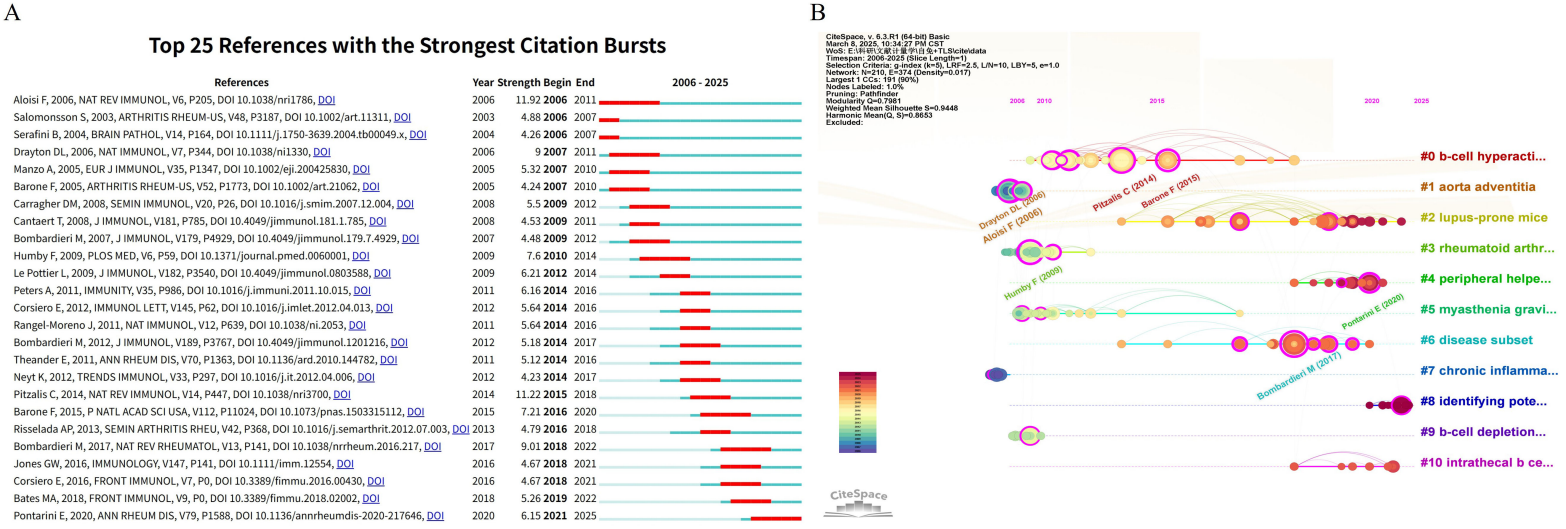
Citation burst analysis and co-cited reference clustering timeline. **(A)** Top 25 references with the strongest citation bursts from 2006 to 2025. Red bars indicate the time period during which a reference experienced a citation burst. **(B)** Timeline view of co-cited reference clusters, showing the temporal evolution of major knowledge domains in the field.

As shown in **Figure 5A**, burst detection of cited references further revealed stage-specific shifts in the focus of knowledge development in this field. Early burst references were mainly concentrated on the work of Aloisi, Salomonsson, Drayton, Manzo, and Barone, indicating that early studies focused on the formation of ectopic lymphoid structures, chronic inflammatory microenvironments, and classical autoimmune pathological mechanisms. More recent burst references were more frequently associated with authors such as Bombardieri, Corsiero, Bates, and Pontarini, and some bursts persisted until 2025, indicating that current research frontiers have gradually shifted toward disease subtype identification, functional stratification of B cells, and the exploration of novel regulatory mechanisms.

The timeline view of co-cited reference clusters is shown in **Figure 5B**. The network clustering showed high quality, with a modularity Q of 0.7981 and a weighted mean silhouette S of 0.9448, indicating good structural stability of the clustering results. According to the clustering labels, the major clusters included b-cell hyperactivity, aorta adventitia, lupus-prone mice, rheumatoid arthritis, peripheral helper, myasthenia gravis, and disease subset. In terms of overall evolution, early studies were mainly centered on classical themes such as lymphoid neogenesis, chronic inflammation, and rheumatoid arthritis. Subsequently, research gradually expanded to myasthenia gravis, B-cell depletion therapy, and organ-specific immune injury. More recently, the focus has further shifted toward lupus-prone models, peripheral helper T cells, disease heterogeneity, and the identification of potential mechanisms. These findings suggest that the intellectual evolution of this field has not advanced along a single disease line, but has instead continuously refined and expanded around the main axis of “tertiary lymphoid structures–aberrant B-cell activation–disease-specific immune microenvironment.”

### 3.6 Keyword clustering, hotspot distribution, and frontier evolution

**Figure 6:**
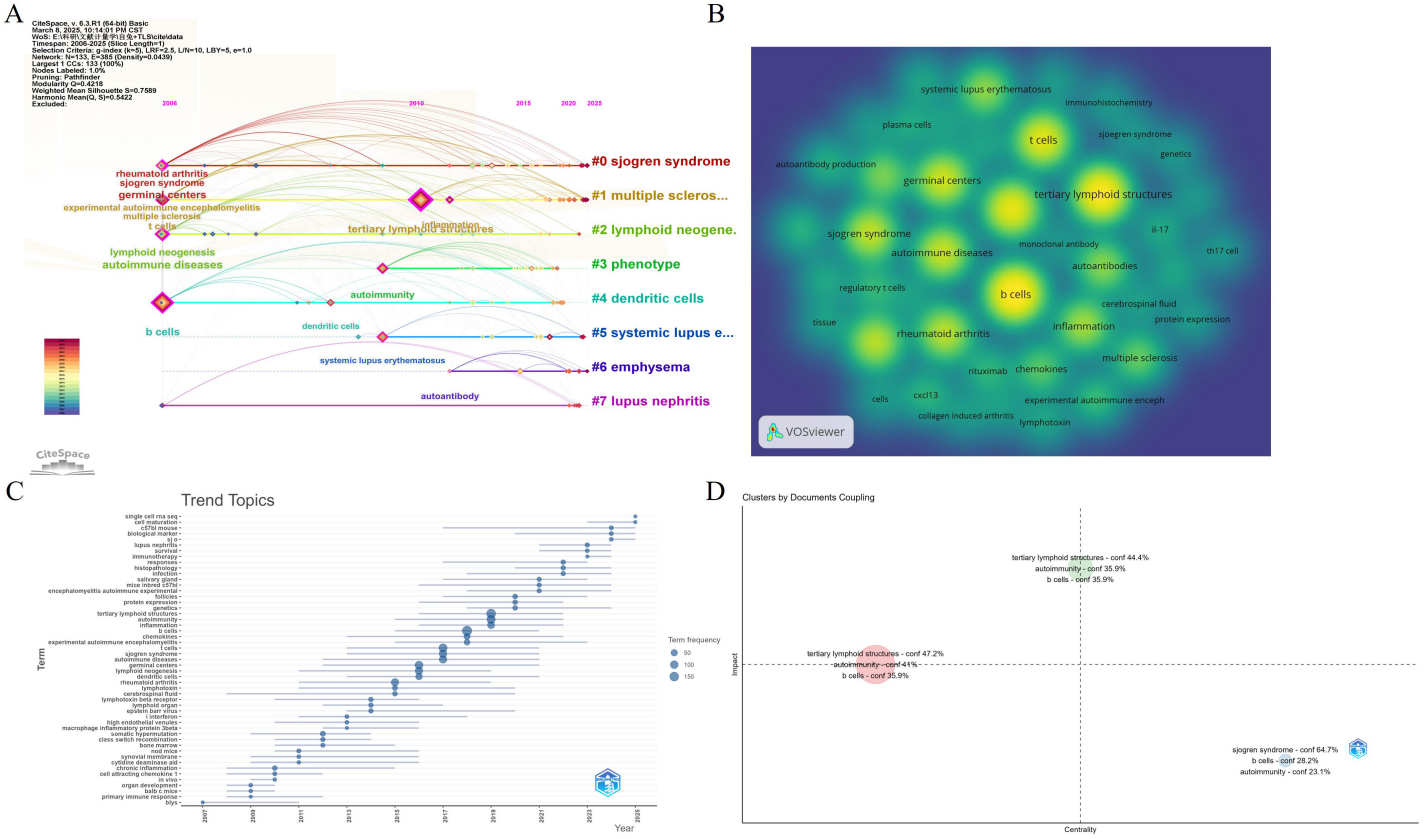
Keyword clustering, hotspot distribution, and thematic evolution in research on tertiary lymphoid structures in autoimmune diseases. **(A)** Timeline view of keyword clusters showing the temporal evolution of major research themes. **(B)** Keyword density map highlighting the most intensively studied topics. **(C)** Trend topics analysis showing the appearance and persistence of major terms over time. Bubble size represents term frequency. **(D)** Document coupling cluster map showing the centrality and impact of major thematic domains.

As shown in **Figure 6A**, the keyword timeline clustering formed a relatively continuous thematic evolutionary structure, and the overall clustering quality was acceptable (Q = 0.4218, S = 0.7589). The major clusters included Sjogren syndrome, multiple sclerosis, lymphoid neogenesis, phenotype, dendritic cells, systemic lupus erythematosus, emphysema, and lupus nephritis. Overall, early studies focused more on rheumatoid arthritis, Sjögren’s syndrome, germinal centers, and lymphoid neogenesis, and then gradually extended to disease contexts such as systemic lupus erythematosus and multiple sclerosis, before further shifting toward phenotypic stratification and organ-specific immune injury. This pattern suggests that keyword evolution in this field has progressed from shared mechanisms toward disease-specific expansion. At the same time, the results were highly consistent with the co-citation clusters, both indicating that TLSs and autoimmune-related immune microenvironments represent the core research direction of this field. Keyword analysis further illustrated, at the thematic level, the extension of this main axis across different disease settings.

The keyword density map is shown in **Figure 6B**. High-density regions were mainly concentrated on terms such as tertiary lymphoid structures, b cells, t cells, germinal centers, autoimmunity, autoantibodies, and inflammation, indicating that TLSs and the associated B-cell/T-cell immune responses remain the central focus of this field. Meanwhile, keywords such as Sjogren syndrome, systemic lupus erythematosus, rheumatoid arthritis, CXCL13, IL-17, and rituximab were located around the hotspot areas, suggesting that current research has increasingly linked structural immune microenvironments with specific disease phenotypes, cytokine axes, and targeted therapies.

The trend topics analysis is presented in **Figure 6C**. Earlier high-frequency topics mainly involved lymphoid neogenesis, dendritic cells, rheumatoid arthritis, and chronic inflammation, whereas more recent topics gradually included lupus nephritis, biological marker, cell maturation, and single-cell RNA sequencing. This shift indicates that the research focus is moving from traditional histopathological and inflammatory mechanism descriptions toward the analysis of cellular heterogeneity, biomarker identification, and refined stratification.

As shown in **Figure 6D**, the document coupling cluster analysis demonstrated that tertiary lymphoid structures, autoimmunity, and b cells formed multiple high-confidence clusters and occupied central positions in the knowledge structure of this field. Among them, Sjogren syndrome also showed high clustering confidence, suggesting that it has become an important disease context linking structural research with clinical application.

## 4 Discussion

### 4.1 Overall evolutionary characteristics of TLS research in autoimmune diseases

The bibliometric findings of the present study indicate that research on TLSs in autoimmune diseases has shown sustained growth, with a particularly marked acceleration after 2015. As reflected by the annual publication trend, co-citation clusters, and keyword timeline analysis, the intellectual development of this field has consistently revolved around three closely connected themes: lymphoid neogenesis, B-cell responses, and disease-specific immune microenvironments. At the same time, the research focus has progressively shifted from the early recognition of ectopic lymphoid structures and their morphological characteristics toward functional interpretation, disease heterogeneity, and potential therapeutic relevance.

From the perspective of thematic evolution, the early phase of the field was mainly devoted to establishing the conceptual basis of TLSs. Aloisi et al. proposed that organized lymphoid structures resembling secondary lymphoid organs can arise in chronically inflamed target tissues, a process described as “lymphoid neogenesis,” and emphasized that this phenomenon should not be regarded as a simple accumulation of inflammatory cells, but rather as a dynamic process of tissue organization involving B-cell follicles, germinal centers, and high endothelial venules (16). Drayton et al. further placed TLSs within a continuum from “ontogeny” to “neogenesis,” thereby providing a developmental framework for understanding how such structures emerge in non-lymphoid tissues under inflammatory conditions (17). Consistent with these landmark studies, the early co-citation structure identified in the present analysis was largely centered on lymphoid neogenesis and classical autoimmune inflammatory settings, indicating that the initial stage of TLS research was primarily concept-building and mechanism-defining.

The second phase was characterized by a shift from structural identification to functional validation. In line with the highly cited references and major co-citation clusters observed in the present study, the most influential publications in this period focused less on the mere presence of TLSs and more on whether these structures could sustain local immune reactions. Bombardieri et al. explicitly proposed that ectopic lymphoid structures in rheumatic autoimmune diseases are not only morphologically organized, but may also display functional ectopic germinal center features, thereby contributing to the maintenance of disease-specific autoantibody production and potentially influencing disease severity and treatment response (18). This interpretation corresponds closely to the co-citation clusters identified in Section 3.5, such as “b-cell hyperactivity,” “b-cell depletion,” and “disease subset,” and helps explain why B cells, germinal centers, and disease stratification have remained central to the intellectual structure of the field.

More recently, the field has entered a stage of refined mechanistic investigation. Dong et al. noted that TLSs are widely present under chronic inflammatory conditions and that, in autoimmune diseases, they are more often linked to uncontrolled local immunity and tissue injury than to protective immune effects (19). This view is highly consistent with the keyword clustering and trend-topic analyses in Section 3.6, which showed a shift from earlier themes such as “lymphoid neogenesis,” “dendritic cells,” and “rheumatoid arthritis” toward more resolved topics including “phenotype,” “disease subset,” “peripheral helper,” and single-cell sequencing. Along the same line, Nayar et al. used single-cell transcriptomics, spatial transcriptomics, and proteomics to characterize TLSs in Sjögren’s syndrome at molecular and spatial levels, further identifying fibroblast states and pericyte/mural cell states with immune-organizing functions (20). Taken together, these findings suggest that TLS research in autoimmune diseases has broadly evolved through three consecutive stages: an initial concept-defining stage, a function-validating stage centered on local B-cell responses and autoantibody generation, and a refined mechanistic stage marked by single-cell, spatial, and cell-interaction-based analyses. In this process, TLSs have gradually shifted from being regarded as a histological phenomenon to becoming an important entry point for understanding local immune amplification, disease heterogeneity, and potential precision-targeted intervention in autoimmune diseases.

### 4.2 Core knowledge base and landmark publications in TLS research

The highly cited references, locally highly cited references, and co-citation clustering results of the present study collectively indicate that the knowledge base of this field was not formed in a fragmented manner, but was progressively established around a limited number of key publications. These studies successively addressed a series of central questions, including what TLSs are, whether they possess local immunological function, and how they participate in disease stratification and progression. They also constitute the major intellectual foundation underlying the co-citation knowledge base identified in Section 3.5.

Aloisi et al. (16) represent one of the most foundational contributions in this field. This study not only proposed that organized structures resembling secondary lymphoid organs can arise in chronically inflamed target tissues, but more importantly suggested that these structures may support B-cell maturation, somatic hypermutation, and class-switch recombination, thereby shifting TLSs from being viewed as a purely histological phenomenon to being regarded as functional units capable of sustaining local immune responses. Drayton et al. (17) further placed TLSs within a continuum “from ontogeny to neogenesis” from the perspective of lymphoid organ development, thereby providing a more comprehensive theoretical framework. Consistent with the early co-citation structure identified in the present study, these two landmark publications established the conceptual basis of TLS research and remained central intellectual anchors of the field over a prolonged period.

At the functional level, Humby et al. (21) demonstrated in RA synovium that ectopic lymphoid structures can sustain AID expression and support local ACPA production and immunoglobulin class switching, even in the absence of newly recruited immune cells. This finding represented a major turning point in the field, indicating that TLSs are not passive bystanders of inflammation, but rather local niches capable of continuously amplifying autoimmune responses. Similarly, Bombardieri et al. (22) showed in the salivary glands of SjD that AID was expressed not only in regions associated with follicular dendritic cell networks, but also in interfollicular large B cells, further supporting the existence of local B-cell expansion and maturation within target organs. Chang et al. (23) extended this concept to lupus nephritis by demonstrating that tubulointerstitial inflammation can also organize into T:B-cell aggregates and germinal center-like structures, accompanied by local B-cell clonal expansion and somatic hypermutation. At the same time, Le Pottier et al. (24) further reported that truly classical germinal center-like structures with canonical GC phenotype and AICDA expression are uncommon in the salivary glands of SjD, and that most B-cell aggregates are not typical germinal centers, although they may still contain autoreactive B cells. Together, these studies suggest that the interpretation of TLSs should not remain limited to whether they are present, but should further incorporate their degree of maturation, cellular composition, and actual functional state.

At the mechanistic level, Manzo et al. (25) and Barone et al. (26) demonstrated in RA synovium and SjD salivary glands, respectively, that CXCL13 and CCL21 are closely associated with the progressive organization of TLSs, and that these chemokines already appear at early aggregation stages, suggesting that they may precede T/B segregation and follicular dendritic cell network formation. Subsequently, Barone et al. (27) proposed that IL-22 can promote CXCL12/CXCL13 expression and drive TLO assembly, whereas Rangel-Moreno et al. (28) showed that IL-17 participates in ectopic lymphoid tissue formation. This line of evidence is highly consistent with the clustering patterns observed in Sections 3.5 and 3.6 around lymphoid neogenesis, B-cell hyperactivity, and related inflammatory pathways, indicating that TLS research has progressed from the recognition of structural presence to the elucidation of mechanisms underlying TLS formation and maintenance.

Over the past decade, the focus of influential publications has further shifted toward disease stratification and clinical relevance. Bombardieri et al. (18) systematically proposed that TLSs not only contribute to the maintenance of autoantibody responses, but may also correspond to distinct disease phenotypes and influence disease severity as well as therapeutic response. Dennis et al. (29) reported that different synovial phenotypes in RA are associated with differential responses to biologic therapies, suggesting that TLS research has extended from pathological observation to disease stratification. More recently, Pontarini et al. (30), Nayar et al. (20), and Masuo et al. (31) have further delineated the complex interactions among helper T-cell populations, stromal cells, and B cells within TLSs from the perspectives of Tfh/Tph biology, spatial omics, and cellular subset analysis. Consistent with the later-stage emergence of themes such as “disease subset,” “peripheral helper,” and “single-cell RNA sequencing” in the keyword analysis of the present study, these publications indicate that TLS research is moving beyond classical structural and functional interpretation toward spatially resolved analysis, cell-level mechanistic identification, and precision-targeted intervention.

Overall, the major publications in this field outline a clear intellectual trajectory: Aloisi and Drayton defined the conceptual basis of TLSs (16, 17); Humby, Bombardieri, Chang, and Le Pottier established their local immune function and heterogeneity (21–24); Manzo, Barone, and Rangel-Moreno clarified key mechanisms involved in TLS formation and maintenance (25–28); and Bombardieri, Dennis, Pontarini, Nayar, and Masuo further advanced the field toward disease stratification, spatial analysis, and precision-targeted intervention (18, 20, 29–31). These developments indicate that although the central intellectual axis of TLS research has remained stable, the key questions driving the field have progressively evolved from concept establishment to mechanistic analysis and clinical translation.

### 4.3 From structural description to functional interpretation: deepening and shifting of research themes

The keyword clustering, co-citation clustering, and timeline analyses of the present study indicate that the evolution of TLS research did not simply reflect thematic expansion, but rather a clear shift from structural identification to mechanistic interpretation of tissue organization. Early studies mainly focused on whether locally organized aggregates resembling secondary lymphoid organs were present in chronically inflamed tissues and whether these structures exhibited features such as T/B-cell compartmentalization, high endothelial venules, and follicular dendritic cell networks (16, 17). On this basis, subsequent studies further showed that the *in situ* expression of chemokines such as CXCL13 and CCL21 in RA synovium and SjD salivary glands was closely associated with the transition of TLSs from loose aggregates to organized structures, and that these molecules often appeared before full TLS maturation, suggesting that they are not merely accompanying phenomena but may participate in the early establishment and maintenance of TLSs (25, 26). Thus, a major advance at this stage was that TLSs were no longer regarded as static pathological features of chronic inflammation, but were instead understood as dynamic organizational processes driven by local chemokine networks.

The research focus subsequently shifted from how TLSs form to whether TLSs truly exert immunological functions. Humby et al. demonstrated in RA synovium that TLSs can sustain AID expression and support local ACPA production and immunoglobulin class switching (21). Bombardieri et al. further showed in the salivary glands of SjD that AID is expressed not only in regions associated with follicular dendritic cell networks but also in interfollicular large B cells, suggesting that local B-cell activation and maturation are not entirely dependent on classical germinal center-like structures (22). Chang et al. extended this concept to lupus nephritis by showing that tubulointerstitial inflammation can organize into T:B-cell aggregates and germinal center-like structures, accompanied by local B-cell clonal expansion and somatic hypermutation (23). At the same time, Le Pottier et al. (24) emphasized that not all morphologically germinal center-like structures share the same functional status. Accordingly, the interpretation of TLSs should not remain limited to their mere presence, but should further incorporate their degree of maturation, cellular composition, and actual function. It was at this stage that TLS research gradually moved beyond pathological morphology toward a functional explanation centered on local autoantibody generation and the maintenance of immune responses.

More recently, the field has entered a phase of refinement and stratification. The later-stage keywords and clustering results of the present study indicate that research hotspots are shifting from traditional themes such as lymphoid neogenesis and dendritic cells toward higher-resolution topics such as disease subset, peripheral helper, and single-cell RNA sequencing. Consistent with this trend, Corsiero et al. showed that B cells differentiated within RA synovial TLSs can recognize NET-associated citrullinated antigens (32). Pontarini et al. reported that the Tfh/Tph cell axis, ICOS, and IL-21 are closely associated with TLSs and MALT lymphoma in SjD (30), whereas Masuo et al. further subdivided Tph cells in RA into stem-like and effector subsets, suggesting distinct layers of helper T-cell function inside and outside TLSs (31). In addition, Nayar et al. and Wu et al. revealed, through spatial omics and single-cell approaches, respectively, the complex interactions among fibroblasts, pericyte-like populations, GZMK^+^ CD8 T cells, and local B-cell responses in SjD and lupus nephritis (20, 33). These findings indicate that current TLS research is no longer confined to local lymphocyte aggregation per se, but is gradually giving rise to a new conceptual framework in which stromal cells, T cells, and B cells jointly shape the local immune niche.

### 4.4 Research focus and shared mechanisms across different disease settings

Consistent with the co-citation clusters, keyword clustering, and author–keyword knowledge structure identified in the present study, TLS research in autoimmune diseases has not evolved as a collection of isolated disease-specific observations, but rather through progressive refinement across several dominant disease models. Among these, RA, SjD, SLE/LN, and MG constitute the principal disease contexts in which TLS research has been developed. As reflected by the repeated occurrence of terms such as “rheumatoid arthritis,” “Sjogren syndrome,” “systemic lupus erythematosus,” “lupus nephritis,” and “myasthenia gravis” in Sections 3.5 and 3.6, these diseases not only provide representative target-organ environments for TLS formation, but also drive the field from asking whether TLSs form to clarifying how TLSs acquire disease-specific functional relevance. In this sense, the development of TLS research in autoimmunity has largely proceeded through disease-stratified comparison of key mechanisms, landmark studies, and pathogenic outputs.

In rheumatoid arthritis (RA), TLS research emerged earliest and has been the most systematic, thereby serving as the most important conceptual and methodological testing ground in this field. The early key theme in RA was the progressive organization of lymphoid-like structures within the synovium. Manzo et al. showed that CXCL13, CCL21, and related chemokine networks are closely associated with TLS formation in rheumatoid synovitis (25), thereby establishing a mechanistic basis for local tissue organization. Humby et al. subsequently demonstrated that synovial TLSs in RA can express AID and support local ACPA production and immunoglobulin class switching (21), clearly defining TLSs in RA as microenvironments with local autoantibody-generating capacity. Corsiero et al. further showed that B cells differentiated within RA synovial TLSs frequently recognize NET-derived citrullinated histones (32), thereby directly linking TLSs to local autoantigen sources and affinity maturation of B cells. More recently, the field has moved toward stratification: Dennis et al. proposed distinct synovial phenotypes, including lymphoid, myeloid, low-inflammatory, and fibroid subtypes, which are associated with therapeutic response (29), whereas Masuo et al. further subdivided Tph cells in RA into stem-like and effector subsets and showed that the former are mainly located within TLSs and provide B-cell help (31). Thus, the RA trajectory can be summarized as a progression from synovial organization and local antibody generation to an integrated framework involving autoantigen-driven responses, T-cell help, and synovial phenotyping.

Compared with RA, TLS research in Sjögren’s syndrome (SjD) has placed greater emphasis on the relationship between TLSs, marked B-cell hyperactivity in target organs, and lymphoproliferative risk. In this disease, the central issue has not simply been whether TLSs are present, but rather how TLS maturation is coupled to glandular B-cell pathology. Nocturne et al. pointed out that SjD is one of the most representative autoimmune diseases characterized by aberrant B-cell activation, and that the local glandular microenvironment not only supports sustained B-cell activation but is also closely associated with the development of MALT lymphoma (34). At the level of landmark studies, Barone et al. and Bombardieri et al. demonstrated that the expression of CXCL13 and CCL21 in salivary glands is closely associated with progressive TLS organization in SjD, and that AID can be detected in regions associated with follicular dendritic cell networks as well as in interfollicular large B cells, suggesting that local B-cell maturation and expansion are continuously sustained within the glands (22, 26). At the same time, Le Pottier et al. proposed that not all morphologically GC-like structures represent true “real GCs” with classical GC function, and that most B-cell aggregates may instead represent different maturation stages while still containing autoreactive B cells (24). This observation is particularly important because it suggests that TLSs in SjD are better understood as a continuum of maturation rather than as a binary presence-or-absence phenomenon. More recently, Pontarini et al. linked Tfh/Tph cells, ICOS, and IL-21 to TLSs and MALT lymphoma in SjD (30), whereas Nayar et al. delineated the spatial interaction network of TLS-associated fibroblast and pericyte-like populations in SjD using single-cell and spatial omics approaches (20). Therefore, SjD most clearly illustrates how TLSs may progress from chronic inflammatory organization to lymphoproliferative disease under conditions of persistent local B-cell hyperactivity.

In systemic lupus erythematosus and lupus nephritis (SLE/LN), TLS research has focused more strongly on local adaptive immune amplification in the context of organ-specific injury. In contrast to RA and SjD, the central theme in SLE/LN is less the existence of local organization itself than the way in which TLSs become coupled to renal inflammation, tissue injury, and irreversible organ damage. Chang et al. demonstrated that tubulointerstitial inflammation in LN can organize into T:B-cell aggregates or GC-like structures, accompanied by intrarenal B-cell clonal expansion and somatic hypermutation (23). This finding suggests that severe renal injury in LN is not determined solely by circulating immune complex deposition, and that local intrarenal adaptive immune responses are also of major importance. More recently, Wu et al. revealed that active LN kidneys exhibit a pronounced extrafollicular B-cell response, characterized by the enrichment of ABCs and ASCs, local clonal expansion, and a low somatic hypermutation rate, together with local accumulation of GZMK^+^ CD8 T cells that spatially colocalize with B cells within TLSs (33). Compared with RA and SjD, this suggests that TLSs in LN may not only support GC-like B-cell responses, but may also be linked to more extrafollicular modes of local B-cell differentiation. Accordingly, TLS research in SLE/LN places greater emphasis on the pathological sequence connecting local tissue injury, adaptive immune amplification, and progressive organ dysfunction.

Myasthenia gravis (MG) represents another organ-specific context of TLS research, but one centered on the thymic microenvironment rather than on joints or exocrine glands (35). Unlike the other disease models, the critical issue in MG is not primarily sustained local autoantibody production within peripheral target tissues, but how a specialized immune organ becomes a site of local tolerance breakdown and autoimmune initiation. Matsui et al. reviewed the strong association between MG and thymic abnormalities, particularly thymic follicular hyperplasia and microenvironmental remodeling in the setting of thymoma, suggesting that the thymus is not merely a passively affected organ, but may instead serve as an important site of local immune tolerance breakdown and autoantibody production (36). Chung et al. further demonstrated that, in patients with thymoma, the presence of thymic germinal centers is significantly associated with the postoperative development of MG, and that GC presence is an independent risk factor (37). Compared with RA, SjD, and LN, TLS research in MG therefore places greater emphasis on the relationship between organ developmental context, local immune dysregulation, and disease risk prediction, and also indicates that TLSs are not limited to classical inflammatory target organs, but may arise within organs with specialized immune functions.

In addition to these major diseases, central nervous system disorders such as multiple sclerosis (MS) have also provided important extensions to TLS research. Pikor et al. demonstrated that meningeal TLS formation depends on Th17 cells and lymphotoxin signaling and is accompanied by local stromal remodeling (38). More recent studies have further suggested that an elevated meningeal CXCL13:BAFF ratio is associated with TLT formation and gray matter injury (39). Although MS is not the principal disease context of the present study, these findings reinforce an important cross-disease consensus: TLS formation does not depend solely on lymphocyte aggregation, but requires the coordinated participation of local inflammatory signals, stromal remodeling, and lymphoid chemokine networks.

Overall, although TLSs in different disease settings exhibit distinct organ backgrounds and clinical implications, their shared mechanisms are relatively clear. First, they all arise under conditions of persistent antigenic stimulation and chronic inflammation. Second, they all depend on signals such as CXCL13, CCL21, IL-17, IL-22, and lymphotoxin to drive local tissue organization (25–28). Third, they are all closely associated with aberrant B-cell activation, autoantibody production, and the persistence of local adaptive immune responses (21–23, 32). At the same time, their dominant pathogenic outputs differ substantially by disease context: RA emphasizes local autoantigen-driven responses, synovial phenotyping, and Tph stratification; SjD highlights sustained B-cell hyperactivity, TLS maturation continuum, and lymphoproliferative tendency; SLE/LN underscores local tissue injury and extrafollicular B-cell responses; and MG reflects thymic microenvironmental dysregulation and disease risk (29–34, 36, 37). Therefore, although TLSs represent a shared mode of local immune organization across autoimmune diseases, their biological consequences are clearly shaped by disease-specific organ context and pathogenic programs.

### 4.5 Research frontiers: aberrant B-cell activation, disease stratification, and mechanism identification driven by single-cell and spatial omics

The keyword trends, burst references, and representative original studies identified in the present analysis indicate that the frontier of TLS research has shifted away from the earlier questions of whether TLSs form and whether germinal center-like structures are present, toward a deeper focus on which cellular programs, helper axes, and organizational networks determine TLS formation, maintenance, and pathological output under persistent inflammatory conditions. In particular, the emergence of later-stage themes such as “disease subset,” “peripheral helper,” and “single-cell RNA sequencing” suggests that the current frontier is no longer defined by the mere presence of TLSs, but rather by an increasingly refined understanding of their cellular composition, spatial organization, and functional states.

First, the aberrant B-cell activation axis has become one of the central frontiers in current TLS research. Traditionally, B-cell responses within TLSs were largely interpreted as classical germinal center-like processes. However, accumulating evidence suggests that TLS-associated B-cell programs are not restricted to a single pathway, but may involve distinct modes and levels of abnormal activation. In RA, Corsiero et al. showed that B cells differentiated within TLSs can recognize NET-associated citrullinated antigens, indicating that TLSs are not only sites of B-cell maturation but may also serve as local niches for the persistent selection and expansion of pathogenic autoantigens (32). In SLE, aberrant B-cell responses and epitope spreading have likewise been recognized as major drivers of sustained autoantibody production (40). In LN, Wu et al. further demonstrated, through single-cell transcriptomics, a pronounced extrafollicular B-cell response characterized by the enrichment of ABCs and ASCs, local clonal expansion, and a relatively low somatic hypermutation rate, indicating that TLS-associated B-cell activation is not limited to the conventional germinal center pathway (33). Thus, current research no longer simply asks whether B-cell activation occurs within TLSs, but increasingly seeks to define which B-cell programs predominate, whether they are germinal center-like or extrafollicular in nature, and how these differences shape local immune amplification and disease-specific injury.

Second, helper T-cell programs, particularly the Tfh/Tph axis, are becoming a key mechanistic framework for understanding functional stratification within TLSs. Pontarini et al. reported in SjD that Tfh/Tph cells, ICOS, and the IL-21 axis are closely associated with TLS formation and MALT lymphoma, suggesting that sustained local B-cell activation is not a spontaneous process but depends on long-term support from specific helper T-cell programs (30). Masuo et al. further subdivided Tph cells in RA into stem-like and effector subsets, showing that the former are mainly localized within TLSs and possess stronger B-cell helper capacity, whereas the latter are more broadly distributed in inflammatory regions (31). Meanwhile, Sowerby et al. emphasized that Tph cells are not merely the peripheral counterparts of Tfh cells, but rather an important functional T-cell population that links CXCL13 production, lymphoid aggregation, and local B-cell help in chronically inflamed tissues (41). Together, these studies suggest that the frontier of helper T-cell research in TLS biology is moving beyond generalized descriptions of T-cell infiltration toward a more mechanistic understanding of which helper programs sustain B-cell responses and how these programs are functionally stratified.

Third, with the development of single-cell and spatial omics technologies, the stromal/fibroblast–CXCL13-related organizational network has emerged as another critical frontier for understanding TLS stability and pathological output. Denton et al. demonstrated that type I interferon can induce CXCL13 expression and thereby support ectopic germinal center formation, suggesting that CXCL13 is not merely a chemokine, but also an important molecular axis linking inflammatory signaling to local tissue organization (9). Zubkova et al. further proposed, from the perspective of artificial TLSs and mesenchymal stromal cell-based platforms, that stromal cells themselves may serve as essential structural scaffolds for immune niche formation (10). In disease-related studies, Hovd et al. showed that podoplanin-expressing macrophages are associated with TLSs (42), whereas Nayar et al. used single-cell transcriptomics, spatial transcriptomics, and proteomics to identify fibroblast states and pericyte/mural cell states closely associated with TLS formation and maintenance, further indicating that stromal cells are not merely passive background components, but key determinants of TLS spatial architecture and functional stability (20). The distinct synovial phenotypes and differential therapeutic responses described by Dennis et al. also support the concept that local microenvironmental states themselves may carry stratifying significance (29). In this sense, the mechanistic gain provided by single-cell and spatial omics lies not simply in identifying more cell types, but in determining which cellular subsets truly participate in TLS organization, which occupy key spatial positions, and which local niche states are more likely to correspond to specific pathological outputs.

On this basis, the potential translational implications of single-cell and spatial omics are also becoming increasingly apparent. Although current evidence is still insufficient to conclude that a specific TLS state can already serve as a directly actionable clinical biomarker, these technologies have at least provided a basis for three important directions. First, they may support more refined disease stratification, by distinguishing local immune niches that are characterized by heightened B-cell activation, dominant helper programs, or enhanced stromal organization. Second, they may contribute to more specific risk assessment, for example, by linking TLS-related structures with MALT lymphoma tendency or associating thymic germinal centers with post-thymectomy MG risk (30, 37). Third, they may facilitate the identification of more precise therapeutic targets, including aberrant B-cell programs, the Tfh/Tph–ICOS–IL-21 axis, and CXCL13-related organizational networks (9, 30, 41). Therefore, the real value of the current single-cell and spatial omics frontier lies not only in improving our understanding of TLS complexity, but also in helping identify which TLS states may be clinically meaningful and which cellular axes may represent candidates for future precision intervention.

Overall, the current frontier of TLS research in autoimmune diseases can be summarized into three interrelated mechanistic directions: first, moving beyond the classical germinal center framework to reinterpret the diverse programs of local aberrant B-cell activation; second, understanding how Tfh/Tph-centered helper programs sustain and shape local B-cell responses; and third, defining TLS spatial organization and functional stability through stromal cells, fibroblasts, and CXCL13-related networks. It is along these converging, cross-disease and cross-cellular mechanisms, together with the continued advancement of single-cell and spatial omics technologies, that TLS research has truly progressed from conventional structural observation to the frontier of mechanistic identification, disease stratification, and precision-oriented therapeutic exploration.

### 4.6 International collaboration patterns and the role of core research forces in driving field development

The country-, institution-, and author-level collaboration networks identified in the present study indicate that TLS research has not developed in a uniformly distributed manner, but rather through a typical pattern of “core countries–core institutions–core teams.” At the national level, the United States consistently occupied the center of the international collaboration network, while several European countries, particularly the United Kingdom, France, Germany, and Italy, formed the most important collaborative circle in this field. At the institutional level, Queen Mary University of London, the University of Birmingham, and their collaborating institutions remained key nodes in the network. At the author level, a relatively stable and continuously productive research community, represented by Bombardieri, Pitzalis, Barone, Humby, Corsiero, and Pontarini, emerged as a major driving force. These findings suggest that major advances in this field have not arisen in isolation, but have instead accumulated progressively through sustained collaborative structures.

Among these collaborative forces, the contribution of UK-based teams has been particularly systematic and best illustrates the continuity from concept establishment to mechanistic refinement and, ultimately, translational relevance. The research trajectory centered on Pitzalis, Bombardieri, Barone, and Humby successively defined the local organizational features of TLSs, their AID-related functions, chemokine networks, and the potential for local autoantibody production in RA and SjD, and later extended these observations to NET-related autoantigen recognition, Tfh/Tph-mediated B-cell help, and microenvironmental dissection using single-cell and spatial omics approaches (20, 30, 32). This continuity is especially important because TLS research inherently spans pathology, tissue immunology, rheumatology, and translational medicine; without long-term accumulation by stable research teams, it is difficult to move from descriptive observation to mechanistic interpretation and clinical relevance.

At the same time, other research groups have expanded TLS research into additional disease settings and technological directions. Neuroimmunology studies represented by the Gommerman group extended the TLS concept to meningeal inflammation and central nervous system injury, emphasizing the roles of Th17 cells, lymphotoxin, and local stromal remodeling in TLS formation (38, 39). Research represented by the Ueno/Yoshitomi group further refined the classification and spatial distribution of Tph-cell subsets in RA, thereby advancing TLS research into the stage of functional subset stratification (31). Meanwhile, teams focusing on LN and MG expanded the organ-specific significance of TLSs from the perspectives of local renal immunity and the thymic microenvironment, respectively (33, 36, 37). Taken together, these diverse contributions indicate that the collaborative structure of the field has not only supported productivity, but has also shaped the expansion of TLS research from a few classical autoimmune disease models toward a broader framework spanning organs, diseases, and technological platforms.

### 4.7 Strengths, limitations, and future perspectives of the present study

Using a bibliometric approach, the present study systematically examined TLS research in autoimmune diseases from multiple dimensions, including countries, institutions, authors, co-cited references, keyword clustering, and temporal evolution, thereby providing a relatively comprehensive overview of the knowledge structure, core themes, and emerging frontiers of this field. In particular, by integrating highly cited references, locally highly cited references, and representative recent original studies into the interpretation, the present work moved beyond a simple description of quantitative distribution and network relationships and, to some extent, delineated the evolutionary trajectory of the field from structural identification to mechanistic analysis and from classical pathological observation to single-cell- and spatial omics-based investigation. The major strength of this study therefore lies in combining bibliometric findings with key original evidence, thereby enhancing the interpretability of the developmental trajectory of the field rather than merely providing formal visualization.

Nevertheless, several limitations should be acknowledged. First, bibliometric analysis is inherently dependent on database coverage, indexing rules, and citation information. Although the combined use of WoSCC and Scopus improved retrieval breadth, database-specific inclusion bias may still have influenced the final dataset. Second, only English-language publications were included, which may have introduced language bias and led to the underrepresentation of relevant studies published in other languages. Third, citation-based analyses are inevitably affected by citation lag; some recent high-quality studies may not yet have accumulated sufficient citations because of their short publication time and may therefore be underrepresented in co-citation networks and burst analyses. Fourth, TLS research itself is characterized by substantial cross-disease, cross-organ, and cross-technology heterogeneity, and inconsistencies in keyword standardization, terminology evolution (e.g., TLS, TLO, and ELS), and author naming preferences may have influenced the clustering results to some extent. Fifth, although the major conclusions of this study were cross-validated at the content level using representative original studies, differences in TLS maturity, functional state, and clinical significance across distinct diseases remain difficult to determine with full precision by bibliometric analysis alone. In other words, bibliometric analysis is better suited to answering where research is concentrated and how it has evolved, whereas questions such as which mechanisms are most important, which TLS states are functionally dominant, and whether TLSs have equivalent pathogenic significance across diseases still require deeper original evidence. Most importantly, bibliometric analysis can identify trends, associations, and influential themes, but it cannot by itself establish causality.

Looking forward, several directions merit particular attention in future TLS research. First, comparative studies across different autoimmune diseases should be further strengthened in order to clarify the shared mechanisms and organ-specific differences of TLSs in RA, SjD, SLE/LN, MG, and other disorders (18, 19). Second, with the continued development of single-cell sequencing, spatial transcriptomics, multimodal proteomics, and cell–cell interaction analyses, TLS research is likely to advance from descriptive characterization of cellular composition toward the identification of dynamic developmental trajectories, spatially organized niche states, and functionally meaningful cellular programs. In particular, the roles of fibroblasts, pericyte-like populations, and Tph/Tfh cells in TLS establishment and maintenance warrant sustained attention (20, 31, 41). Third, future work should place greater emphasis on linking TLS-related molecular and spatial features with disease stratification, therapeutic response, and prognostic assessment, thereby promoting the transition of TLS research from pathology- and mechanism-oriented investigation toward more clinically relevant precision applications (19, 20, 37, 43). Overall, TLSs are no longer viewed merely as structural hallmarks of chronic inflammation, but are increasingly regarded in the literature as important entry points for understanding persistent local adaptive immunity, disease heterogeneity, and potential therapeutic targets. Their translational relevance deserves further in-depth investigation.

## 5 Conclusion

In summary, this bibliometric analysis shows that TLS research in autoimmune diseases has evolved into a relatively mature and continuously expanding field. Research attention has shifted from early structural description toward functional interpretation, disease heterogeneity, and single-cell/spatially resolved investigation. Importantly, the literature increasingly views TLSs as active local immune niches rather than mere histological features of chronic inflammation. Further studies integrating mechanistic investigation with clinical stratification are required to clarify their translational relevance in precision medicine.

## Data Availability

The raw data supporting the conclusions of this article will be made available by the authors, without undue reservation.
